# Predictive Factors Associated With Fear Faced by Healthcare Workers During COVID-19 Pandemic: A Questionnaire-Based Study

**DOI:** 10.7759/cureus.9741

**Published:** 2020-08-14

**Authors:** Jagdesh Kumar, Muhammad Soughat Katto, Adeel A Siddiqui, Badaruddin Sahito, Bashir Ahmed, Muhammad Jamil, Maratib Ali

**Affiliations:** 1 Orthopaedic Surgery, Dow University of Health Sciences, Karachi, PAK; 2 Anaesthesiology, Dow University of Health Sciences, Karachi, PAK

**Keywords:** fear, healthcare worker, covid-19, pandemic

## Abstract

Background

The coronavirus disease 2019 (COVID-19) since the beginning has been a reason of fear among healthcare workers (HCWs) due to the increased mortality, especially in the HCWs themselves. In this survey study, we aimed to explore the predictive factors associated with fear faced by HCWs during the COVID-19 pandemic and to identify the areas which need to be addressed to reduce it.

Methods

On May 14, 2020, we conducted an observational, cross-sectional survey using a self-administered questionnaire, consisting of the following two parts: (1) focused on factors associated with HCWs’ fear of getting an infection and being a source of carrying the infection to whom they care, and (2) focused on factors associated with HCWs’ fear of uncertainty and lack of support from concerned health authorities.

Results

The mean age of the participants was 40.04 ± 12.92 years with 79.3% being males. More than half (51.1%) of the participants were consultants. The most important factors associated with fear included getting infected (84.8%), quarantined (69.6%), not getting medical treatment (62%), losing a life (56.8%), and infecting family members (94.2%). Another major factor associated with HCWs' fear was lack of support from concerned health authorities, 80.2% thought of solatium, and 71.7% believed that the job should be given to eligible family members of the deceased. More than 82.2% were concerned about health expenses and around 97.6% felt an additional health risk allowance should be given.

Conclusion

Our results indicate that the risk of getting infection to themselves and their families, along with a lack of support from concerned health authorities, was strongly associated with fear among HCWs. We hope through these findings that the concerned health authorities will take notice and do something in this regard by developing appropriate policies and measures to make sure that HCWs and their families are cared for if they get infected.

## Introduction

The coronavirus disease 2019 (COVID-19) and its related threat of death have led to significant fears among healthcare workers (HCWs) and their families around the world [1]. No one knows exactly when the things will return to normal, it spreads so fast, and is so devastating that many countries have issued a stay-at-home advisory and have ordered the shutdown of all non-essential businesses, restaurants, social gatherings, and even teaching institutes in an attempt to limit the spread of the infection [2-4].

To date, the World Health Organization (WHO) has reported 5,939,234 confirmed cases and 367,255 deaths across the world. The risk of getting an infection is not only high in healthcare workers (HCWs) working in a hospital setting but also in their family members, particularly in young children and the elderly [1, 5]. Everyone is watching television and social media for the latest news and updates related to the COVID-19 pandemic and wondering “How and when will this be over?”. The latest figures show that thousands of HCWs have so far been infected with COVID-19 while interacting with patients and a large percentage of them are dead or dying [6-7]. As a result of this fear, isolation, and threat of death, many HCWs are now reluctant to work and went into self-isolation to protect either oneself or a loved one. Additionally, the absence of safety measures to limit the self-contamination and viral inhalation outside of health care settings and lack of financial, as well as medical and social support (in case of infection, isolation, or death), can paradoxically enhance COVID-19-related fear. If these factors concerning the HCWs' fear and uncertainty are not resolved properly, it may result in health crises due to a critical shortage of HCWs in the future.

Considering the relevance of all of the above factors, a cross-sectional, web-based survey was carried out, the purpose of which was to explore the predictive factors associated with the fears faced by HCWs during the COVID-19 pandemic and to identify the areas which need to be addressed to reduce it [8].

## Materials and methods

This observational cross-sectional, questionnaire-based survey was carried out by the Department of Orthopaedic Surgery, Dr. Ruth K. M. Pfau Civil Hospital, affiliated to Dow University of Health Sciences, Karachi, Pakistan. The study participants were HCWs, including consultants, medical officers, postgraduate trainees, house officers, and paramedical staff. An informed consent statement to participate in the study was obtained from each respondent and anonymity was assured. The synopsis was reviewed and approved by the Institutional Review Board of the Dow University of Health Sciences, Karachi, Pakistan.

A convenient sampling method was used and a minimum sample size of 310 was calculated, considering anticipated fear of getting the COVID-19 infection to oneself and loved ones as 72% [3], precision as 5%, and confidence interval (CI) as 95%.

An online self-administered questionnaire with a consent statement for participation was designed on Google survey forms [8]. The HCWs were approached through a link shared via email, as well as through social media platforms using smartphones. The survey was open for five days (May 14 - 18, 2020) to achieve the desired limit. The questionnaire was developed by the investigators with the help of previous literature on COVID-19 fear in HCWs, in addition to basic demographic data (age, gender, job designation, workplace), consisting of two parts: (1) focused on factors associated with HCWs' fear of getting an infection and being a source of carrying the infection to those whom they care for, and (2) focused on factors associated with HCWs' fear of uncertainty and lack of support from concerned health authorities in case they or their family members become infected, isolated, or lose their life [2-3, 9]. For valid and accurate responses, each question was labeled with three choices (Yes; No; Maybe). We did not use a Likert-type scale in our questionnaire because it is often very hard to interpret ambiguous responses from average responses on a Likert scale. Finally, since the minimum sample size was kept at 310, a total of 329 completely filled responses were received in the specified duration.

The information obtained from the participants was coded, validated, and analyzed using the IBM Statistical Package for the Social Sciences (SPSS) Statistics for Windows, Version 22.0 (IBM Corp., Armonk, NY, USA). Mean with standard deviations were calculated for age and frequency with percentages for categorical variables.

## Results

Out of a total of 329 responses, 261 were males (79.3%) and 68 were females (20.7%), with a mean age of 40.04 ± 12.92 years. These gender differences may be due to the higher male enrollment in our institution. By designation, 168 (51.1%) were consultants, 61 (18.5%) were medical officers, 50 (15.2%) were paramedical staff, 35 (10.6%) were postgraduate trainees, and only 15 (4.6%) were in the house officers category. Among these, 214 (65%) participants were from a government setup and 115 (35%) from a private work setting.

When the fear was assessed, 84.8% of participants were afraid of getting infected, 65% were reluctant to work, and 70.5% felt nervous when talking to patients in close vicinity. Around 69.6% were afraid of getting quarantined, whereas 66.3% were anxious about the cost of treatment. When asked if they felt afraid when they hear a colleague was infected and on a ventilator because of COVID-19, most participants (91.2%) answered yes. When asked if they were afraid of getting infected and not getting any medical treatment, a considerable number of participants (62%) responded yes. About 25.2% had sleeping difficulty, 56.8% were afraid of losing their life, and 49.2% were generally furious, angry, and depressed. On evaluating the fear of carrying the infection, most participants (94.2%) were afraid of transmitting the infection to their family members and friends, while 83.9% felt nervous when talking to their family members in close vicinity. When the study participants were asked, "in case of death, do you think that solatium should be given to the family members of the deceased?", 80.2% replied yes and 84.2% thought of the health expenses. A total of 71.7% believed that the job should be given to eligible family members of the deceased and 97.6% thought of an additional COVID-19 allowance. Approximately 88.1% of participants were feeling that health education and training in infection control measures should be given to HCWs before recruitment in COVID-19 centers to prevent the spread of infection and maintain good hygienic practices, along with appropriate personal protective equipment (PPE).

**Table 1:** Predictive Factors Associated With the Fear Amongst Healthcare Workers (n = 329)

Questions	Yes (%)	No (%)	May be (%)
Are you afraid of getting infected with COVID-19 at work?	84.8%	4.6%	10.6%
Are you afraid to go to work because of the risk of exposure?	65%	18.5%	16.4%
Do you feel nervous when talking to patients in close vicinity?	70.5%	15.5%	14%
Are you afraid of getting quarantined from family?	69.6%	12.5%	17.9%
Are you afraid because of not having proper protective equipment at your workplace?	70.2%	14.6%	15.2%
Are you anxious about the cost of treatment if you get infected?	66.3%	19.1%	14.6%
Are you afraid, when you hear colleagues were infected or on a ventilator because of COVID-19?	91.2%	3.6%	5.2%
Do you have a sleeping problem because you’re worried about getting COVID-19?	25.2%	59%	15.8%
In case if you developed COVID-19 symptoms, are you afraid that you may not get medical treatment?	62%	17%	21%
Does the idea of COVID-19 infection make you feel furious, angry, or depressed?	49.2%	29.8%	21%
Are you afraid of losing life because of COVID-19?	56.8%	18.5%	24.6%
Are you afraid to infect your family members and friends?	94.2%	1.2%	4.6%
Are you feeling nervous when talking to your family members in close vicinity, especially with kids and elderly people?	83.9%	6.7%	9.4%
Do you think that solatium should be given to the family members of the deceased?	80.2%	4%	15.8%
In case if you or family members will be infected, do you think that concerned health authorities should pay their health expenses?	84.2%	10.3%	5.5%
Do you think that health education and training in infection control measures should be given to healthcare workers before recruitment in COVID-19 centers, along with appropriate protective gear?	88.1%	9.7%	2.1%
Do you think that job should be offered to the eligible family members of the deceased?	71.7%	9.4%	18.8%
Do you think that health authorities should pay additional health risk allowance to frontline healthcare workers?	97.6%	1.2%	1.2%

## Discussion

The current rapid spread of infection and its related mortality affecting millions of HCWs throughout the globe has lead to a panic mode in the healthcare settings [10-12]. The previous global analysis suggested a higher overall incidence of COVID-19 infection in HCWs than the general population [1, 5]. The current study evaluated the predictive factors associated with the fear faced by HCWs during the COVID-19 pandemic and identifies the areas which need to be addressed. Our findings are consistent with the high level of fear in HCWs during the COVID-19 pandemic. In this study, the most important factors to consider were HCWs’ fears of getting infected (84.8%), quarantined (69.6%), and losing a life (56.8%). Once diseased, a considerable number of participants (62%) feared that they may not get medical treatment and end up on a ventilator. As a result of this excessive fear, around 65% of HCWs were reluctant to work while 70.5% found it difficult to interact with the patients in close vicinity. Additionally, approximately 25.2% of participants had sleep problems, while 49.2% looked angry and depressed.

Indeed, previous research has found that HCWs are afraid of getting infected and quarantined [2]. Certain specific reasons for their fears were family and childcare responsibilities during times of self-isolation or quarantine, risk of carrying the infection to loved one, and lack of specific treatment and vaccine [2]. Moreover, the unavailability of PPE and seeing/hearing of colleagues getting infected and being on a ventilator were also significantly associated with a fear of getting infected [3, 13]. However, because of excessive fear and apprehension of the spread of infection, many of the governments, and even private hospitals, have closed the doors of their outpatient clinics, providing emergency treatment only in an attempt to limit the spread [2]. There are reports from several institutions across Pakistan, as well as from abroad, about HCWs refusing to report for duty because of the lack of protective gear [14-15]. Lack of protective gear not only put HCWs at risk of getting infected but also their families [3, 16]. It is the responsibility of the government and the healthcare institutions to provide adequate protective clothing and equipment. The published data suggest that excessive risk and exposure can be reduced by using appropriate precautions at work, i.e., careful PPE donning and doffing, hand hygiene, social distancing, and cleaning within healthcare settings [5]. If these issues are not identified and resolved appropriately, along with the economic impact, it may lead to a critical shortage of HCWs and health crises.

In this study, another major factor of fear of the COVID-19 was the perceived risk of carrying the infection to loved ones (94.2%), particularly the elderly people at home. Studies have further indicated that HCWs who have children and family members with pre-existing medical conditions showed a higher level of fear [16-17]. Due to this, most of the participants (83.9%) felt nervous when talking to their family members and friends in a closed vicinity. The available data suggests that excess risk and exposure can be minimized by using appropriate precautions at home, i.e., washing hands, change out of their work clothes and showering, gargling with warm salty water, steam inhalation, do a bit of judicious social distancing, particularly with kids and elderly people, avoid sharing utensils, and use separate plates/cups/bowls [2].

In this study, the last and most important factor associated with fear was uncertainty and lack of support from concerned health authorities in case the HCWs or their family members are infected, isolated, or lose a life. Under the Workers' Compensation Act, the employer is responsible to provide cash and medical benefits to workers who are injured or become ill in the course of their employment and provide cash benefits to the survivors of workers killed on the job, even if the worker accepted the known risks of the job [18]. In this study, 80.2% of participants thought that solatium should be given to family members of the deceased, 84.2% worried about health expenses, 71.7% said eligible family members of the deceased should be offered jobs, while 97.6% felt there should be an additional health risk allowance. Approximately 88.1% of participants felt that health education and training in infection control measures should be given to HCWs before recruitment in COVID-19 centers to prevent the spread of infection and maintain good hygienic practices, as well as providing the appropriate protective gear.

This uncertainty that their organization will not support/take care of their personal and family needs if they develop this deadly virus in the line of duty also put one’s mind in fear [4]. If these factors associated with HCWs’ fear are not identified and resolved appropriately, it may result in another, and even worse, health workforce crisis.

Based on the above findings, we recommend that HCWs should be provided with the necessary PPE and material resources required to protect oneself from COVID-19, as well as an additional health risk allowance. Health education and training in infection control measures should be given to HCWs before recruitment in COVID-19 centers to prevent the spread of infection and maintain good hygienic practices. Health and life insurance coverage should be given to HCWs before recruitment for jobs in government, as well as the private sector, in case of the death of HCWs, along with solatium, and the job should be offered to eligible family members of the deceased. If HCWs get infected and hospitalized, the concerned health authorities should pay their health expenses. If HCWs get infected and quarantined, the concerned health authorities should support/take care of their personal and family needs.

This study has several limitations. First, we conducted a questionnaire-based survey method where the data obtained was based on participants' honesty, memory, and ability to respond. Second, the study was of short duration and small sample size. Despite these limitations, we hope attention to these issues may help in alleviating fear faced by HCWs.

## Conclusions

Our results indicate that the risk of getting infection themselves and to their families, along with a lack of support from concerned health authorities, was strongly associated with fear among HCWs. We hope through these findings that the concerned health authorities will take notice and do something in this regard by developing appropriate policies and measures to make sure that HCWs and their families are cared for if they get infected.
